# Simon on Artificial Dilatation of the Anus

**Published:** 1873-06

**Authors:** 


### Simon on the Artificial Dilatation of the Anus and Rectum, for Exploration and for Operation.

Dr G. Simon (*Archiv fur Klinische Chirurgie*, vol. xv. part I. 1872) has shown that it is possible to introduce the whole hand even into the male rectum, and to explore and perform operations without inflicting any injury on the walls of the bowel. The dilatation may be effected with or without inciscion of the sphincters. In the adult, under chloroform, by gradual dilatation, the fingers, half the hand, and then the entire hand and forearm may be introduced if there be no obstruction in the pelvis. In most cases, if the hand does not exceed 9½ inches in circumference, there will not be occasioned more than a slight tearing of the anus, and only very exceptionally a rupture of some of the fibres of the sphincter. When the hand has penetrated the rectum as far as the sacral promontory, three or even four fingers may be carried on up the sigmoid flexure, and then, through the walls of the gut, the whole abdominial region as far as the kidney and the umbilicus can be examined without danger. Thus a more precise diagnosis may be made of affections of the uterus, ovaries, and even the stomach and spleen. With the introduction of only half the hand, the base of the uterus and even the ovaries can be reached; in men the bladder can be felt with precision, and the presence of calculi, their number and size, can be ascertained.

In two cases of ovarian cyst, Dr. Simon was able to determine, by this method of examination, the length and thickness of the pedicle, the absence of adhesions in the pelvic cavity, and lastly, the presence in the fundus of the uterus of two fibrous growths, each as large as a cherry-stone. The operation allowed the verification of this diagnosis. Dr. Simon thinks that full dilatation by the whole hand should be employed in many affections of the rectum; it permits the ready removal of foreign bodies, and in ulceration of the rectum it favors cure by allowing a free discharge of all matters; in fistula it permits the introduction of a speculum analogous to that of Marion Sims, so that internal openings can be seen, and operations upon fistulæ, situated high up the bowel, can be performed with certainty.—*London Medical Record.*

